# The Combined Delivery of the *Vegf*, *Ang*, and *Gdnf* Genes Stimulates Angiogenesis and Improves Post-Ischemic Innervation and Regeneration in Skeletal Muscle

**DOI:** 10.3390/cimb46080507

**Published:** 2024-08-05

**Authors:** Igor Valerievich Samatoshenkov, Alexander Maazovich Aimaletdinov, Elena Yurievna Zakirova, Yuri Alexandrovich Chelyshev, Julia Maratovna Samatoshenkova, Marat Salimovich Kadyrov, Evgeny Alekseevich Kniazev, Bulat Ilgamovich Salakhov, Yana Olegovna Mukhamedshina

**Affiliations:** 1 Medizinische Hochschule Brandenburg Theodor Fontane, 14770 Brandenburg an der Havel, Germany; 2 Institute of Fundamental Medicine and Biology, Kazan (Volga Region) Federal University, 420008 Kazan, Russia; allekss1982@mail.ru (A.M.A.); lenahamzina@yandex.ru (E.Y.Z.); yana.k-z-n@mail.ru (Y.O.M.); 3 Department of Histology, Cytology and Embryology, Kazan State Medical University, 420012 Kazan, Russia; chelyshev-kzn@yandex.ru; 4 Republican Clinical Hospital, 420138 Kazan, Russia; julia.samatoshenkova@mail.ru; 5 Limited Liability Company “Angiolife”, 420015 Kazan, Russia; forts75@mail.ru; 6 Artemed Fachklinik, 32545 Bad Oeynhausen, Germany; zemfi2002@mail.ru; 7 Department of Cardiovascular and Endovascular Surgery, Kazan State Medical University, 420012 Kazan, Russia; kzn2512@gmail.com

**Keywords:** chronic lower limb ischemia, rats, *Gdnf*, *Vegf*, *Ang*, adenoviral vector, umbilical cord blood mononuclear cells

## Abstract

In this study, the effects of different combinations of the genes *Vegf*, *Ang*, and *Gdnf* injected both using direct virus-mediated injection (adenovirus, Ad5) and umbilical cord blood mononuclear cells (UCBCs) on the processes of stimulation of post-ischemic innervation, angiogenesis, and regeneration in skeletal muscle were investigated in a rat hindlimb chronic ischemia model. It was shown that more pronounced stimulation of angiogenesis and restoration of post-ischemic innervation were achieved both in the early (28 days post-ischemia, dpi) and late (42 dpi) terms of the experiment in the calf muscle when UCBCs delivered the combination of Ad5-*Vegf* and Ad5-*Ang* compared to the direct injection of the same vector combination into the area of ischemia. At the same time, the inclusion of Ad5-*Gdnf* in the combination of Ad5-*Vegf* and Ad5-*Ang* directly injected or administered by UCBCs provided a significant increase in the number of centronuclear muscle fibers, indicating stimulation of post-ischemic reparative myogenesis. This study allowed us to determine the most effective gene combinations for angiogenesis and neurogenesis, which, in the future, may serve as the basis for the development of gene and gene cell products for the treatment of chronic lower limb ischemia.

## 1. Introduction

Chronic lower limb ischemia is a pressing medical and social issue. Chronic limb ischemia is a very common condition. For example, on the European continent, the prevalence is 5.3%, in relation to the 750 million inhabitants of Europe. In the European Union itself, there are an estimated 17 million patients with lower limb ischemia, which is 3.4% of the EU’s 500 million inhabitants. The total prevalence of the disease worldwide is about 202 million people [1]. Separately, chronic limb ischemia is closely associated with diabetes mellitus and often leads to limb amputations [2,3].

Conservative treatment of this condition is often ineffective, and many patients have limitations to limb revascularization due to distal lesions or severe somatic diseases [4]. The possibility of overcoming post-ischemic disturbances is related to the introduction of angiogenesis stimulators into the area of ischemia [5]. The most studied angiogenesis stimulators are injected into the ischemic area to restore blood flow in the limb in experiments. It was found that combinations of angiogenic factors work better than the factors administered separately, suggesting a synergistic effect. Several clinical studies have shown the angiogenic effects of virus-mediated delivery angiogenin (*Ang*) and vascular endothelial growth factor (*Vegf*) which have improved muscle perfusion, increased pain-free walking distance, reduced the recovery time of baseline blood flow parameters, and improved patients’ quality of life [5,6].

Interest in the role of innervation in the development of ischemia and overcoming its consequences has been around for a long time [7]. However, the few rigorous confirmations of the influence of nerves on ischemic tissue have been made recently. In one, the combination of limb ischemia with denervation showed a decrease in capillary density and endothelial cell proliferation and increased secretion of nerve growth factor (NGF) and VEGF on day 28 compared to the ischemia group without denervation, indicating the participation of nerve fibers in overcoming the effects of ischemia [8]. One hypothesis explains the change in the vascular tone of denervated arteries. Another hypothesis suggests that one of the functions of sympathetic innervation is to stabilize the phenotype of vascular wall cells, which can lead to thickening of the intima and reduced blood flow [9].

A close histogenetic relationship between nerve fibers and blood vessels has been demonstrated. Nerves and blood vessels follow each other closely, and during formation, they respond to common signals such as semaphorins, netrins, and ephrins, which stimulate the growth of nerve fibers and blood vessels. The observed deterioration of tissue repair in ischemia may also be due to impaired nerve fiber function caused by the ischemia itself. Sensitive nerve fibers are the most vulnerable and are damaged in ischemia first, compared to motor fibers [10]. Ischemia reduces the rate of impulse conduction along sensitive nerve fibers [11]. If we consider that angiogenesis is impaired during arterial denervation, the application of neurotrophic factor genes encoding the synthesis of glial neurotrophic factor (GDNF) and/or NGF, which support neuronal survival and nerve fiber growth, may stimulate restoration of blood flow in the limb.

Currently, gene and gene cell therapies are considered the most promising methods for stimulating angiogenesis, neurogenesis, and regeneration of skeletal muscle in limb ischemia. Since VEGF and ANG are well-known stimulators of angiogenesis, their use in ischemic tissue injury is justified. The role of the neurotrophic component in these processes should be considered. It is recommended to include GDNF in the therapeutic strategy due to its neurotrophic effect and direct influence on ischemic skeletal muscle and target cells in the vessel wall [12,13]. Previously, it was found that when embryonic stem cells of amniotic fluid were transduced with an adenoviral vector carrying the *Gdnf* gene, they expressed the endothelial cell marker von Willebrand factor and CD31 and were able to differentiate into endothelial cells in vitro. Recombinant GDNF stimulated this process [12]. GDNF has a proangiogenic effect, activating endothelial cell proliferation by binding to receptors of neurotrophic factors, tropomyosin receptor kinases A and B, GFRα1, and c-Ret [13]. The effect of GDNF on the activation of the STAT3 signaling pathway, which is a direct transcriptional activator of VEGF, has been shown [14].

From the perspective of choosing a cell source for gene cell therapy in skeletal muscle ischemia, the use of umbilical cord blood seems promising. The absence of legal, ethical, and religious prohibitions associated with umbilical cord blood mononuclear cell (UCBC) transplantation is also an important factor. This population of cells is able to secrete numerous growth and trophic factors and can produce specialized cells from various tissues, stimulating angiogenesis [15,16]. Therefore, it becomes clear from the above-mentioned studies that the combination of angiogenic and neurotrophic factors may be the most effective for overcoming the consequences of skeletal muscle ischemia and requires further research.

Currently, there are two main methods for introducing genetic constructs: virus-mediated direct gene therapy and gene cell therapy. The method of direct gene delivery involves delivering them directly into damaged tissue cells. Cell transplantation, or gene cell therapy, is another approach for stimulating angiogenesis in chronic ischemia of the lower extremities. Cell transplantation is safe and effective in treating ischemic lesions of the lower extremities and has been shown to increase not only blood flow but also the formation of new blood vessels [17].

The aim of this work is to determine the most effective combination of genes administered directly or with stem cells to stimulate angiogenesis, neurogenesis, and skeletal muscle regeneration. In this study, the effects of various combinations of angiogenic and neurotrophic factors (VEGF, ANG, GDNF) on the processes of post-ischemic innervation, stimulation of angiogenesis, and regeneration in ischemic skeletal muscle were investigated. The results obtained may serve as a basis for developing innovative drugs for treating conditions associated with circulatory insufficiency and the need to enhance reparative processes in skeletal muscle tissue.

## 2. Materials and Methods

### 2.1. Creation of Adenoviral Vectors

To obtain recombinant Ad5-*Vegf*, Ad5-*Ang,* and Ad5-*Gdnf* adenoviruses, an adenoviral vector plasmid was linearized using the restriction enzyme PacI. The purified linear plasmid was used to genetically modify HEK293A cells using TurboFect transfection reagent (Thermo Scientific, Waltham, MA, USA). After transfection, the medium was replaced every 2–3 days with fresh medium until cytopathic changes in cell morphology appeared. On day 10 after transfection, cell suspensions were collected into 2 mL sterile tubes and subjected to several freeze/thaw cycles and then centrifuged to obtain crude viral lysate. The viral stock was stored at −80 °C. To obtain preparative amounts of adenoviral vectors, HEK293A cells were infected with crude viral lysate. After 72 h, cell lysates were collected in 15 mL tubes and subjected to several freeze/thaw cycles, and then centrifuged to obtain viral stock. The supernatant was filtered and then purified using two rounds of gradient cesium chloride centrifugation, dialyzed against 50 mM Tris-HCl, pH 7.5, 150 mM NaCl, and then titrated according to the manufacturer’s recommendations for the pAd/CMV/V5-Dest system (Invitrogen, Carlsbad, San Diego, CA, USA).

### 2.2. Isolation and Genetic Modification of Human Umbilical Cord Blood Mononuclear Cells

This study, including the collection of mononuclear blood cells from the human umbilical cord, was approved by the local ethics committee of Kazan State Medical University (excerpt from the minutes of meeting no. 2 of 20 February 2018). Cord blood was collected after informed consent of the pregnant woman and prenatal screening for contraindications to blood donation. All blood manipulations were performed in the laboratory of Kazan Federal University.

Nucleated blood cells were isolated in 50 mL tubes. Each tube was filled with 25 mL of Ficoll solution (PanEco, Moscow, Russia) with a density of 1.077 g/mL, to which an equal volume of cord blood with anticoagulant (the blood and anticoagulant ratio ranged from 1:1 to 3:1) was carefully added using an automatic dispenser. Blood was centrifuged at 720× *g* for 20 min and clear separation of blood into 4 fractions was obtained: erythrocytes, Ficoll, leukocytes, and plasma. The leukocyte fraction was taken in a separate tube, resuspended in sterile 1:2 DPBS solution, and centrifuged at 305× *g* for 15 min. The resulting cell sediment was resuspended in 10 mL of DPBS solution and centrifuged at 305× *g* for 15 min. To remove red blood cells, cells were resuspended in hypotonic lysis buffer (0.168 M NH_4_Cl, 0.1 M KHCO_3_, 1.27 mM EDTA pH 7.3) and, in the final step, cells were washed with DPBS solution. Blood mononuclear fraction cells, after isolation, were kept in low-adhesion dishes (d = 10 cm) with RPMI-1640 medium supplemented with 10% FBS and a mixture of penicillin and streptomycin antibiotics (100 U/mL; 100 μg/mL) (PanEco, Moscow, Russia).

Human cord blood mononuclear cells were genetically modified with recombinant adenoviruses Ad5-*Vegf*, Ad5-*Ang*, and Ad5-*Gdnf* immediately after cell isolation according to the protocol described previously [18]. Cells were infected with previously dialyzed adenovirus with a viral particle titer of 10 (MOI 10). After the virus addition, cells were maintained in RPMI1640 + 10% FBS solution for 16–18 h in a CO_2_ incubator with 5% CO_2_. Subsequently, the cells were washed with DPBS and resuspended in a physiological solution.

### 2.3. Modeling of Ischemia in the Hindlimb

The experiments were carried out on 100 white pubescent laboratory rats of the Wistar line (Pushchino, Moscow, Russia), females and males, weighing 200–250 g. Rats were anesthetized with a Telazol/Xyla mixture (Zoetis, Parsippany, NJ, USA). The position of the animal on the operating table was lying on the back, and the upper and lower extremities were fixed. In the area of the operating field on the inner surface of the thigh of the left hindlimb, the hair was shaved. Ischemia of the limb was created by placing two ligatures made of non-absorbable Ethicon Prolene thread (40) (Miami, FL, USA), 3 mm apart in the femoral artery with a diameter of 2 mm (Supplementary Figure S1) [19]. The area between the ligatures was sectioned. Visual control of hemostasis was performed. The wound was sutured with Ethicon Vicril (30) absorbable suture material. Animals received a single injection of 1 mL of ceftriaxone antibiotic diluted in physiological solution (Borisov Plant for Medical Preparations, Belarus) into the thigh muscle of the contralateral limb immediately after surgery to prevent postoperative infectious complications. To determine the occurrence of ischemia, the intensity of blood flow was measured using an Easy LDI microcirculation measuring device (Aïmago SA, Lausanne, Switzerland). Measurements expressed in absolute perfusion units (apu) were performed before surgery, on the next day, and on the fifth day after surgery. In addition, calf muscle was collected from the control group of animals for histological analysis 1 day after ischemia was created.

**Figure 1:**
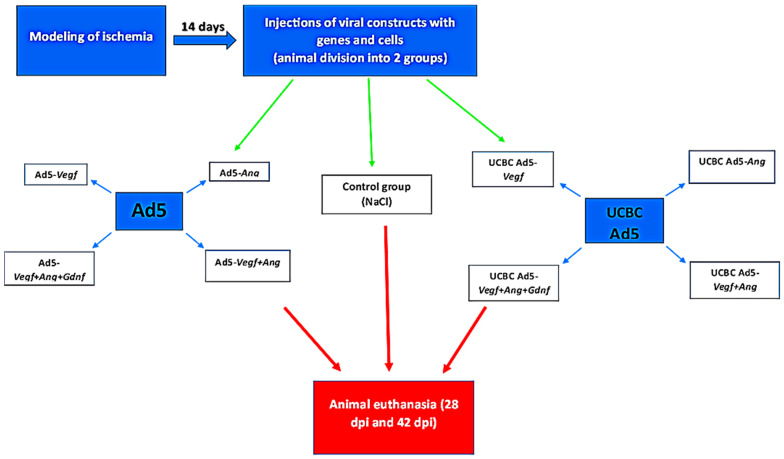
Schematic of the experiment. At 14 days after modeling ischemia, animals were injected with adenoviral vectors or UCBCs at 4 points in the distal part of the calf muscle. On 28 and 42 days after modeling ischemia (dpi), the animals were euthanized.

On 14 and 28 days after the surgical intervention to create hindlimb ischemia, rats were injected with gene or gene cell constructs into the distal part of the calf muscle at 4 points. On 28 and 42 days after modeling ischemia, the animals were euthanized (Figure 1).

The experiments were carried out in full compliance with the ethical principles established by the European Convention for the Protection of Vertebrate Animals Used for Experimental and Other Scientific Purposes (adopted in Strasbourg on 18 March 1986 and confirmed in Strasbourg on 15 June 2006).

### 2.4. Experimental Groups

Fourteen days after the creation of hindlimb ischemia, the animals were randomly divided into 2 experimental groups: group 1—with direct virus-mediated gene injection (n = 40) and group 2—with cell-mediated gene delivery using UCBCs (n = 40). Each of these experimental groups included 4 subgroups with intramuscular injection of different genetic constructs or their combinations in the variant of direct or cell-mediated delivery. Furthermore, a control group (n = 10) was formed, whose animals were injected with 0.9% NaCl (Dalchimpharm, Russia, Moscow) under similar experimental conditions.

Animals in experimental group 1 were injected with adenoviruses carrying different combinations of genes—Ad5-*Vegf* (n = 10), or Ad5-*Ang* (n = 10), or Ad5-*Vegf* + Ad5-*Ang* (n = 10), or Ad5-*Vegf* + Ad5-*Ang* + Ad5-*Gdnf* (n = 10) by 2 × 10^10^ virus particles in 60 μL of 0.9% NaCl in the distal part of the calf muscle at 4 points of 15 μL each.

Animals in experimental group 2 received a similar injection of UCBC transduced adenoviruses—UCBC Ad5-*Vegf* (n = 10), or UCBC Ad5-*Ang* (n = 10), or UCBC Ad5-*Vegf* + Ad5-*Ang* (n = 10), or UCBC Ad5-*Vegf* + Ad5-*Ang* + Ad5-*Gdnf* (n = 10) by 2 × 10^6^ cells in 60 μL of 0.9% NaCl. For primary screening of the expression of genes encoding recombinant proteins, additional experimental groups of animals were formed, which were injected intramuscularly with Ad5-*Egfp* (n = 5) or UCBC Ad5-*Egfp* (n = 5) under similar experimental conditions on the 14th day after modeling ischemia.

Twenty-eight and forty-two days after modeling ischemia, the animals were euthanized. For this purpose, the animals were anesthetized and transcardially perfused with a 4% paraformaldehyde solution (4 °C).

### 2.5. Histological and Immunohistochemical Methods

For analysis, the distal part of the calf muscle was sampled and embedded in paraffin using standard methods. Transverse muscle slices, 5 µm thick, were prepared on a microtome (PFM Medical GmbH Rotary 3002, Berlin, Germany) and used for subsequent morphometric and immunohistochemical analysis.

For the morphometric analysis of muscle fibers and the counting of centronuclear muscle fibers (CNMFs), hematoxylin staining of transverse sections was used (BioVitrum, Saint Petersburg, Russia). To evaluate ischemic skeletal muscle damage, hematoxylin and eosin-stained muscle tissue was microscopically evaluated. Assessment of capillary density and count of the number of CNMFs were performed in the area of ischemia and at a distance within 500 μm from the area of insertion of the genetic constructs. Morphometric analysis was performed using Image Scope software (Aperio ImageScope x64 1.50i).

Immunofluorescence analysis using CD31 antibodies was performed in transverse sections of the calf muscle (Table 1). Donkey secondary antibodies against rabbit IgG conjugated to Alexa Fluor 555 were used for visualization. The nuclei were stained with DAPI (Lumiprobe, Moscow, Russia). Fluorescence images were obtained using a laser confocal microscope, LSM 700 (Carl Zeiss, Jena, Germany), at a magnification of ×630. To identify nerve fibers, immunohistochemical analysis was performed with antibodies against the Schwann cells marker protein S100b and the axon marker β3-tubulin. The application of antibodies to the slices was carried out after dehydration and dewaxing of the slices followed by antigen demasking in Tris-EDTA (pH = 9.0, Merck, Darmstad, Germany). Antigen detection was carried out using the Novolink system (Leica Biosystems, Nussloch, Germany). Visualization was performed with aminoethylcarbazole chromogen. The nuclei were stained with hematoxylin. Slices were encapsulated under coverslips using glycerogel (Tverskaya Pharmafabrik, Tver, Russia). The light images were digitized using an Aperio CS2 scanner (Leica Biosystems, Nussloch, Germany).

### 2.6. Statistical Processing

Statistical analysis and visualization of the data obtained were performed using the R 3.6.3 statistical computing environment (R Foundation for Statistical Computing, Vienna, Austria). Descriptive statistics for quantitative variables are presented as mean (standard deviation) and median (1st and 3rd quartiles). In the comparison of the studied groups, one-factor analysis of variance and the Kruskal–Wallis test were used; Welch’s t-test and Dunn’s test were used as post hoc methods, respectively; differences were considered statistically significant at *p* < 0.05.

## 3. Results

In the hindlimbs of the rats, a persistent decrease in blood flow by 45% was recorded immediately after surgical intervention and by 41% (from baseline) 5 days after the ischemia creation. After surgery, blood flow in the operated limbs decreased significantly from 123 ± 0.8 to 56 ± 0.5 apu. Subsequently, blood flow values remained stable at 62 ± 1.3 apu until the introduction of genetic constructs on the 14th day after the creation of ischemia (see Supplementary Figure S2).

**Figure 2:**
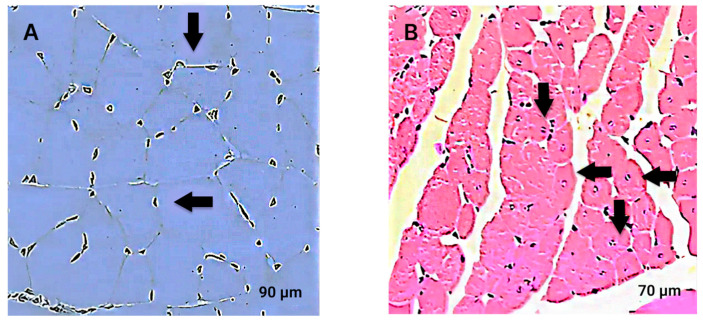
Distal part of the calf muscle in different terms after modeling ischemia. (**A**)—1 day after the creation of ischemia, ischemic damage to the muscle in the form of minor destruction of muscle fibers (arrows), cross section. (**B**)—14 days after the creation of ischemia, muscle fibers with disintegrated sarcoplasm disappear, transverse striation disappears, numerous CNMFs (arrows) appear, cross section. Hematoxylin and eosin staining. Light microscopy.

Histological analysis of the calf muscle of the operated limb on the first day showed insignificant ischemic changes. The number of capillaries in contact with one muscle fiber was noted to range from 2 to 5. The muscle fibers completely preserved their characteristic structure. CNMFs were present in an insignificant amount. On the 14th day after the creation of ischemia, pronounced ischemic changes were observed. The muscle was infiltrated with polymorphonuclear leukocytes, muscle fibers with eosinophilic and disintegrated sarcoplasm were present, some muscle fibers showed a loss of transverse striation and lymphohistocytic infiltration, and destructive muscle tissue was replaced by connective tissue in a significant volume. There was a decrease in the density of the capillaries surrounding the muscle fibers and also the presence of CNMFs (Figure 2A,B).

### 3.1. Expression of Egfp upon Intramuscular Administration of Ad5-Egfp and UCBC Ad5-Egfp 

**Figure 3:**
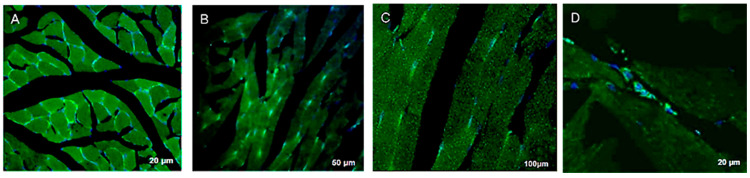
Distal part of the calf muscle at a distance of 500 μm from the injection area at 14 days after injection of Ad5-*Egfp* (**A**–**C**) or UCBC Ad5-*Egfp* (**D**) into the muscle. (**A**) Transverse slice; EGFP luminescence (green) contours the muscle fiber profile. (**B**,**C**) Lengthwise section; EGFP luminescence in the form of clusters distributed along the length of the fiber. (**D**)Lengthwise section; EGFP luminescence in cells in contact with muscle fibers. Nuclei stained with DAPI (blue). Confocal microscopy.

On the 28th day of the experiment, in the Ad5-*Egfp* injection group of animals (14 days after injection), specific glowing EGFP was observed in the distal part of the calf muscle, which corresponded to injection points. The glow was most intense in the periphery of muscle fibers near the sarcolemma and in the region of muscle fiber nuclei (Figure 3A–C).

On the 28th day of the experiment, in the group of animals with UCBC Ad5-*Egfp* transplantation, the presence of these cells in the injected area and their efficient expression of EGFP were established (Figure 3D). The data obtained indicate that the activity of genetic constructs with transgene expression is maintained for at least 14 days when Ad5-*Egfp* or UCBC Ad5-*Egfp* is injected into the area of ischemia.

### 3.2. Assessment of Capillary Density in the Area of Ischemia of the Experimental Groups

**Figure 4:**
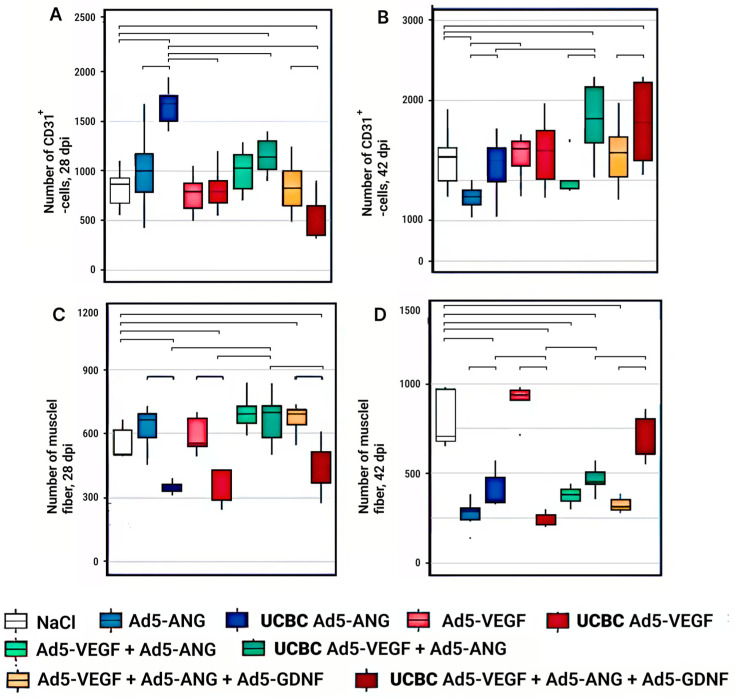
Number of CD31+ cells (**A**,**B**) and normal muscle fibers (**C**,**D**) at 28 and 42 dpi in the experimental groups. Groups with significant differences are combined by horizontal lines with serifs (*p* < 0.05).

On the 28th day, in experimental group 1, the highest capillary density, assessed by the number of CD31+ cells, was recorded in the Ad5-*Vegf* + Ad5-*Ang* subgroup (Figure 4A). However, significant differences were not found between other experimental groups with the direct introduction of genetic constructs and the control group. During the same time period, with cell-mediated gene delivery, the maximum capillary density was observed in the UCBC Ad5-*Ang* group. In the UCBC Ad5-*Ang* group, the capillary density in the distal part of the calf muscle was 2.0 times (*p* < 0.05) higher than in the control group (NaCl). Thus, 28 days after ischemia modeling (14 days after the moment of gene and gene cell therapy), according to the capillary density criterion, in the calf muscle of the ischemic limb, the expression of this index decreased in the following sequence: UCBC Ad5-*Ang* → UCBC Ad5-*Vegf* + Ad5-*Ang* → Ad5-*Vegf* + Ad5-*Ang* → Ad5-*Ang* → Ad5-*Vegf* + Ad5-*Ang* + Ad5-*Gdnf* → UCBC Ad5-*Vegf* → Ad5-*Vegf* → UCBC Ad5-*Vegf* + Ad5-*Ang* + Ad5-*Gdnf* (Figure 4). It should be noted that significant differences with the NaCl control group were observed only in the UCBC Ad5-*Ang* and UCBC Ad5-*Vegf* + Ad5-*Ang* groups, where the number of CD31+ cells was higher (*p* < 0.05) (Figure S3).

At 42 days after ischemia modeling, the highest capillary density in the calf muscle of the ischemic limb was recorded in the Ad5-*Vegf* group to which gene constructs were administered directly, while the highest capillary density was recorded in the UCBC Ad5-*Vegf* + Ad5-*Ang* group on which cell-mediated gene delivery was performed (Figure 4B).

According to this measure, UCBC-mediated delivery of Ad5-*Vegf* and Ad5-*Ang* was 2.5- and 1.4-fold (*p* < 0.05) more effective (*p* < 0.05) than direct delivery of Ad5-*Vegf* + Ad5-*Ang* or NaCl injection, respectively. Therefore, 42 days after ischemia modeling (28 days from the moment of gene and gene cell therapy), according to the capillary density criterion in the calf muscle of the ischemic limb, the expression of this index decreased in the following sequence: UCBC Ad5-*Vegf* + Ad5-*Ang* → UCBC Ad5-*Vegf* + Ad5-*Ang* + Ad5-*Gdnf* → Ad5-*Vegf* → UCBC Ad5-*Vegf* → Ad5-*Vegf* + Ad5-*Ang* + Ad5-*Gdnf* → UCBC Ad5-*Ang* → Ad5-*Vegf* + Ad5-*Ang* → Ad5-*Ang.* Furthermore, it should be noted that significant differences with the NaCl control group were observed only in the UCBC Ad5-*Vegf* + Ad5-*Ang* and UCBC Ad5-*Vegf* + Ad5-*Ang* + Ad5-*Ang* + Ad5-*Gdnf* groups, where the number of CD31+ cells was higher (*p* < 0.05) (Supplementary Figure S4).

### 3.3. Analysis of Normal Muscle Fibers in the Ischemia Area of the Experimental Groups

According to the criterion of the number of normal muscle fibers at 28 days after ischemia modeling, the most pronounced positive effect was registered in the UCBC Ad5-*Vegf* + Ad5-*Ang* group, where this index was 1.5–2 times (*p* < 0.05) higher compared to other groups where cell-mediated gene delivery was performed (Figure 4C). The expression of the above-mentioned index decreased in the following sequence: UCBC Ad5-*Vegf* + Ad5-*Ang* → Ad5-*Vegf* + Ad5-*Ang*→ Ad5-*Vegf* + Ad5-*Ang* + Ad5-*Gdnf* → Ad5-*Ang* → Ad5-*Vegf* → UCBC Ad5-*Vegf* + Ad5-*Ang* + Ad5 *Gdnf* → UCBC Ad5-*Ang* → UCBC Ad5-*Vegf*. It should be noted that a significant difference was observed with the NaCl control group only in the Ad5-*Vegf* +Ad5-*Ang* + Ad5-*Gdnf* group, where the number of normal muscle fibers was greater (*p* < 0.05).

According to the criterion of maintaining the number of normal muscle fibers at 42 days after ischemia modeling, the most pronounced positive effect was recorded in the Ad5-*Vegf* group, which showed significant differences compared to the NaCl control group (Figure 4D). The *A*d5-*Vegf* group exhibited a 3.3-fold increase in the number of normal muscle fibers compared to the *Ad5-Ang* group (*p* < 0.05). The expression of the above-mentioned index decreased in the following sequence: Ad5-*Vegf* → UCBC Ad5-*Vegf* +Ad5-*ANG* + Ad5-*Gdnf* → UCBC Ad5-*Vegf* +Ad5-*Ang* → Ad5-*Vegf* +Ad5-*Ang* → UCBC Ad5-*Ang* → Ad5-*Vegf* + Ad5-*Ang* + Ad5-*Gdnf* → Ad5-*Ang* → UCBC Ad5-*Vegf*.

### 3.4. Analysis of the Capillary/Muscle Fiber Ratio in the Ischemia Area of the Experimental Groups 

**Figure 5:**
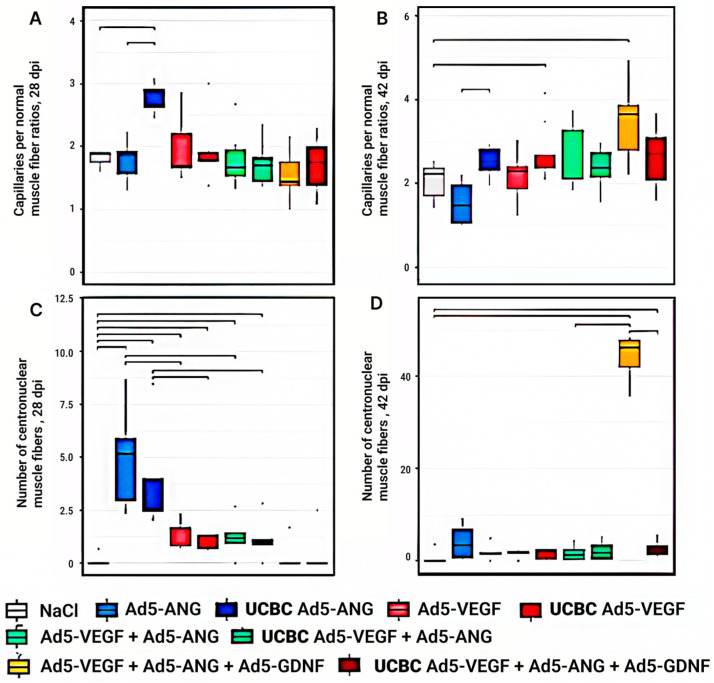
Ratio of capillaries per normal muscle fiber (**A**,**B**) and number of centronuclear muscle fibers (**C**,**D**) at 28 and 42 dpi in experimental groups. Groups with significant differences are combined by horizontal lines with serifs (*p* < 0.05).

At 28 days after the ischemia modeling, the capillaries per normal muscle fiber ratio showed the highest value in the UCBC Ad5-*Ang* group, which showed increases of 1.4- and 1.5-fold (*p* < 0.05) in this index compared to the NaCl control group and the Ad5-*Ang* group (Figure 5A). Forty-two days after modeling ischemia, the highest capillary value per normal muscle fiber ratio was found in the Ad5-*Vegf* + Ad5-*Ang* + Ad5-*Gdnf* group, where there was a significant difference of 1.6 times with the control group. In the UCBC Ad5-*Vegf* group, this index was 1.2 times higher than in the control group. An increase in the value of this indicator by 1.6 times was revealed in the UCBC Ad5-*Ang* group compared to the Ad5-*Ang* group (Figure 5B).

### 3.5. Analysis of CNMF in the Area of Ischemia of the Experimental Groups

According to the CNMF quantity criterion, 28 days after ischemia modeling, the most pronounced effect was recorded in the Ad5-*Ang* and UCBC Ad5-*Ang* groups, where these indices were 37.3 and 30 times higher (*p* < 0.05) in comparison with the NaCl control group, respectively (Figure 5C). The expression of the above-mentioned index decreased in the following sequence: Ad5-*Ang* → UCBC Ad5-*Ang* → Ad5-*Vegf* → Ad5-*Vegf* + Ad5-*Ang* → UCBC Ad5- *Vegf* + Ad5-*Ang* → UCBC Ad5-*Vegf* → UCBC Ad5-*Vegf* + Ad5-*Ang* + Ad5-*Gdnf* → Ad5-*Vegf* + Ad5-*Ang* + Ad5-*Gdnf*.

At 42 days after modeling ischemia, the number of CNMFs was maximal in the Ad5-*Vegf* +Ad5-*Ang* + Ad5-*Gdnf* group, where this index was 81 times higher (*p* < 0.05) when compared to the NaCl control group (Figure 5D). The expression of the above-mentioned index decreased in the following sequence: Ad5-*Vegf* + Ad5-*Ang* + Ad5-*Gdnf* → Ad5-*Ang* → UCBC Ad5-*Vegf* + Ad5-*Ang* + Ad5-*Gdnf* → Ad5-*Vegf* → UCBC Ad5-*Vegf* → UCBC Ad5-*Ang* → UCBC Ad5-*Vegf* + Ad5-*Ang* → Ad5-*Vegf* + Ad5-*Ang*.

### 3.6. Number of S100b+ Cells in the Area of Ischemia in Experimental Groups

**Figure 6:**
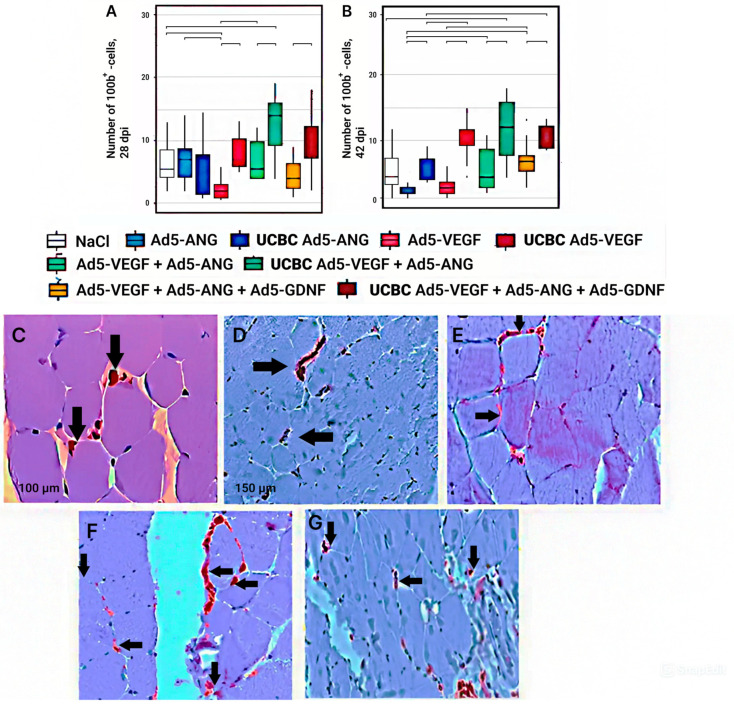
Number of S100b+ cells at 28 dpi (**A**) and 42 dpi (**B**) in the experimental groups. Groups with significant differences are combined by horizontal lines with serifs (*p* < 0.05). Immunohistochemical visualization of S100b+ cells in the following groups at 42 dpi: control (**C**), Ad5-*Vegf* (**D**), Ad5-*Vegf* + Ad5-*Ang* (**E**), Ad5-*Vegf* + Ad5-*Ang* + Ad5-*Gdnf* (**F**), UCBC Ad5-*Vegf* + Ad5-*Ang* + Ad5-*Gdnf* (**G**). Arrows indicate stained nerve fibers.

In terms of the number of S100b+ cells (Schwann cells), maximum values were achieved 28 days after modeling ischemia in the groups with cell-mediated delivery of the genetic constructs UCBC Ad5-*Vegf* + Ad5-*Ang* and UCBC Ad5-*Vegf* + Ad5-*Ang* + Ad5-*Gdnf* (Figure 6A). However, only in the UCBC Ad5-*Vegf* + Ad5-*Ang* group did the number of S100b+ cells have a significant difference (2.5 times) compared to the NaCl control group. The expression decreased in the following sequence: UCBC Ad5-*Vegf* + Ad5-*Ang* → UCBC Ad5-*Vegf* + Ad5-*Ang* + Ad5-*Gdnf* → UCBC Ad5-*Vegf* → Ad5-*Vegf* → Ad5-*Ang* → Ad5-*Vegf* + Ad5-*Ang* → UCBC Ad5-*Ang* → Ad5-*Vegf* + Ad5-*Ang* + Ad5-*Gdnf* (Figure 6A).

At 42 days after modeling ischemia, the highest number of S100b+ cells was registered in the UCBC Ad5-*Vegf* + Ad5-*Ang* group, where this index was 2.4 times higher (*p* < 0.5) in comparison with the NaCl control group (Figure 6B). At the same time, other groups with cell-mediated gene delivery had significant differences in the numbers of S100b+ cells compared to the corresponding groups with direct gene therapy and the control group. The expression decreased in the following sequence: UCBC Ad5-*Vegf* + Ad5-*Ang* → UCBC Ad5-*Vegf* + Ad5-*Ang* + Ad5-*Gdnf* → UCBC Ad5-*Vegf* → Ad5-*Vegf* → Ad5-*Vegf* + Ad5-*Ang* + Ad5-*Gdnf* → UCBC Ad5-*Ang* → Ad5-*Vegf* + Ad5-*Ang* → Ad5-*Ang.*

### 3.7. Number of Nerve Fibers in the Ischemic Area of the Experimental Groups

**Figure 7:**
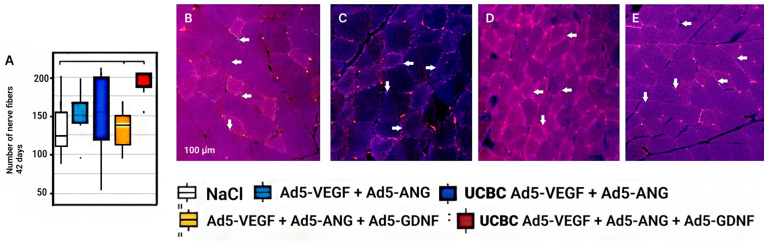
Number of nerve fibers (**A**) at 42 dpi in experimental groups. Groups with significant differences are combined by horizontal lines with serifs (*p* < 0.05). Immunohistochemical visualization of β3-tubulin+ fibers in Ad5- *Vegf* +Ad5-*Ang* (**B**), Ad5-*Vegf* +Ad5-*Ang* + Ad5-*Gdnf* (**C**), UCBC Ad5-*Vegf* + Ad5-*Ang* (**D**), UCBC Ad5-*Vegf* + Ad5-*Ang* + Ad5-*Ang* + Ad5-*Gdnf* (**E**) at 42 dpi in those groups. β3-tubulin+ fibers are indicated by arrows.

The number of nerve fibers analyzed by β3-tubulin expression was assessed on day 42 in the groups with cell-mediated and direct delivery of Ad5-*Vegf* + Ad5-*Ang* or Ad5-*Vegf* + Ad5-*Ang* + Ad5-*Gdnf*. The maximum number of β3-tubulin+ nerve fibers was recorded at the injection site of UCBC Ad5-*Vegf* + Ad5-*Ang* and UCBC Ad5-*Vegf+* Ad5-*Ang* + Ad5-*Gdnf*, which is correlated with the data on the number of S100b+ cells (Figure 7).

## 4. Discussion

To objectively assess the effects of gene and gene cell constructs on angiogenesis and the regeneration of muscle tissue, it is necessary to obtain confirmation of the adequacy of the modeled chronic ischemia of the lower limbs. During the evaluation of the applied model of chronic ischemia of the lower limbs of the rats, we performed an instrumental assessment of the level of blood flow, which confirmed a significant decrease in perfusion in the operated limbs. Surgical ischemia of the limb resulted in the development of typical pathological changes in the state of the transverse striated skeletal muscle tissue. At the same time, we did not observe extreme manifestations of ischemia, gangrene formation, and self-amputation of the limb. This complex of morphological changes in skeletal muscle was stably reproduced in the experiment, which allows us to consider the chosen experimental model as suitable for testing the effect of transgenes on overcoming the consequences of ischemia.

To fully achieve the main therapeutic goals of direct gene and cell-mediated therapy, it is important to maximize the presence of the adenoviral vector or transplanted cells and the angiogenesis and regeneration stimulator genes they deliver in the injured tissue for as long as possible.

The data obtained in this work indicate that human UCBCs transplanted into the area of rat limb ischemia survive for at least 14 days with the possibility of expressing recombinant gene products. On day 14 of the experiment, we observed specific fluorescence in the ischemic muscle after injection of an adenoviral vector containing EGFP. This indicates the expression of the recombinant gene during this period in the case of direct gene therapy. Our data confirm previous studies in which similar adenoviral vectors and transduced cells were injected into other tissues and organs [20,21,22,23]. Research has shown that genetically modified UCBCs can survive for at least 4 weeks under xenotransplantation conditions [22,24]. This suggests that the transplanted cells themselves and the transgenes they express may influence virtually all stages of post-ischemic reparative myogenesis, from the early events immediately after injury to the formation of definitive muscle fibers that restore damaged tissue and skeletal muscle function. 

Although the first signs of post-ischemic muscle regeneration appear within a few hours after the onset of ischemia, this process is prolonged, resulting in a slow recovery of muscle function. In our study, we found that the gene and gene cell constructs we used had different effects on muscle fiber preservation, angiogenesis, and post-ischemic innervation 14 and 28 days after their injection (28 and 42 days after the modeled ischemia). In this respect, it seems more promising to use those approaches that lead to more effective tissue regeneration in the delay period, taking into account the duration of the possible regeneration of muscle tissue and restoration of its functional characteristics.

Chronic ischemia of the lower extremities results in muscle fiber damage and degeneration with overgrowth of connective tissue. The results of our experiments showed no significant changes in the number of preserved muscle fibers in the experimental groups with direct or cell-mediated gene therapy compared to the control group. On the contrary, most of the experimental groups (except UCBC Ad5-*Vegf* + Ad5-*Ang* + Ad5-*Gdnf* and Ad5-*Vegf*) had a reduced number of preserved muscle fibers up to 42 days after ischemia. It can be assumed that this effect is associated with an increase in the number of CNMFs, indicating reparative regeneration of muscle tissue [25,26], in the experimental groups compared to the control group.

However, this possibility is not excluded only for the groups with direct or cell-mediated delivery of the *Vegf*, *Ang*, and *Gdnf* genes, where the number of CNMFs was significantly higher compared to the control group. Taken together, these features suggest that in chronic hypoxic injury, the mechanism of reparative myogenesis is most actively stimulated by a combination of all three transgenes studied.

In general, satellite cells and other myogenic progenitor cells interact closely with endothelial cells during muscle regeneration, which stimulates myogenic cell growth and, conversely, differentiating myogenic cells promote angiogenesis. The results of our experiments showed the greatest positive effect on angiogenesis of the cell-mediated delivery of therapeutic transgenes, where the index of the number of CD31+ cells was highest 42 days after ischemia in groups with the combined delivery of genes encoding angiogenic factors (UCBC Ad5-*Vegf* + Ad5-*Ang*) or their combination with neurotrophic factor (UCBC Ad5-*Vegf* + Ad5-*Ang* + Ad5-*Gdnf*). *Gdnf* plays an important role in VEGF-driven revascularization of ischemic muscle, including endothelial cell outgrowth and vascular maturation [27].

The role of GDNF in endothelial cell adhesion and migration, leading to the restoration of a functional vascular network, may be mediated by the adhesion molecule integrin β1, which together with other partners is involved in signaling through the GDNFRα-1 receptor and is critical for vascular pattern formation and remodeling of the vascular network [28,29].

The low rate and degree of post-ischemic muscle regeneration were not associated with a specific loss of satellite cells, nor with a decrease in capillary density or expression of the main growth factors controlling myogenesis [30]. However, these negative manifestations during reparative myogenesis may be a consequence of the prolonged inflammation and oxidative stress that occur during muscle ischemia and reperfusion. We also evaluated the ratio of capillaries per normal muscle fiber, the highest value of which was observed at 42 dpi in the group with direct delivery of the *Vegf*, *Ang*, and *Gdnf* genes.

In this group, the positive effect of the expression of the VEGF transgene was constantly manifested. The most obvious explanation for the stimulatory effect of VEGF on reparative myogenesis is based on the well-studied effect of this factor on angiogenesis and reperfusion of blood vessels in muscle, leading to improved trophism of muscle tissue. At 42 dpi, the more pronounced effect of VEGF with direct vector injection compared to its cell-mediated delivery may be due to a decrease in the number of genetically modified UCBCs caused by their increasing death rate.

The sympathetic nervous system plays an important role in angiogenesis [31]. Activation or inhibition of adrenergic receptors (mainly β-adrenergic receptors) expressed on endothelial cells and pericytes significantly affects the formation of new blood vessels. According to the criterion of maintaining the number of Schwann cells, cell-mediated delivery was found to be more effective than direct injection of any of the gene combinations studied at 28 and 42 days after ischemia. The maximum number of S100b+ cells was observed in groups with the combined delivery of genes encoding angiogenic factors (UCBC Ad5-*Vegf* + Ad5-*Ang*) or their combination with neurotrophic factor (UCBC Ad5-*Vegf* + Ad5-*Ang* + Ad5-*Gdnf*). When the number of nerve fibers was evaluated at 42 dpi by immunohistochemical reaction with antibodies against β3-tubulin in the Ad5-*Vegf* + Ad5-*Ang*, Ad5-*Vegf* + Ad5-*Ang* + Ad5-*Gdnf*, UCBC Ad5-Vegf + Ad5-*Ang*, and UCBC Ad5-*Vegf* + Ad5-*Ang* + Ad5-*Gdnf* groups, the maximum number of nerve fibers was observed, which corresponds to the data on the number of nerve fibers when stained with antibodies against S100b.

It has previously been shown that exogenous *Vegf* increases *Gdnf* expression in damaged skeletal muscle, which in turn stimulates nerve fiber regeneration and recovery of function [27]. Overexpression of GDNF in skeletal muscle leads to a significant increase in the number of neuromuscular synapses [32]. GDNF not only promotes motor neuron maturation, but also supports regeneration of damaged axons and modulates neuromuscular transmission by acting at both pre- and postsynaptic levels. Consequently, the addition of *Vegf* leads to angiogenic and neurogenic responses that affect axon growth directly or indirectly through increased expression of GDNF by cells of the reorganized vascular network. The expression of these responses may vary depending on the stage of regeneration and the predominance of one or the other component in a particular area of regeneration. Therefore, cell-mediated combined delivery of genes encoding angiogenic and neurotrophic factors to ischemic tissue may have a direct stimulatory effect on nerve fiber regeneration and may also stimulate this process indirectly by enhancing the effect of GDNF. Therefore, the restoration of innervation and perfusion contributes to the normalization of skeletal muscle structure and function in the post-ischemic period, which was most pronounced in the UCBC Ad5-*Vegf* + Ad5-*Ang* + Ad5-*Gdnf* group.

In conclusion, we would also like to note the gradual spread of gene therapy using a combination of different genes in such nosologies as limb ischemia plus diabetes. It was shown that the use of pIRES/VEGF165/HGF plasmid in patients with lower limb ischemia and diabetes significantly improved vascularization of the affected limb [33]. Another study showed enhanced migration of CD34+ cells upon intramuscular administration of plasmids with a combination of ANG1/VEGF genes [34]. The above studies suggest a possible potentiating effect of gene combination compared to monotherapy with a single therapeutic gene.

This study shows that of eight combinations of three transgenes, *Vegf*, *Ang*, and *Gdnf*, injected directly or via human umbilical cord blood mononuclear cells into the region of ischemia, cell-mediated delivery of the combination of Ad5-*Vegf*, Ad5-*Ang*, and Ad5-*Gdnf* stimulates revascularization, reparative myogenesis, and nerve fiber regeneration more effectively up to 42 days after ischemia. The beneficial effects of specific transgenes encoding angiogenic and neurotrophic factors on the regeneration of ischemic skeletal muscle vary depending on the stage of the process.

## Figures and Tables

**Table 1:** Primary and secondary antibodies used in immunohistochemical and immunofluorescence analyses.

Antibody	Host	Dilution	Source
CD31	Rabbit	1:150	Abcam (Cambridge, UK)
Alexa Fluor 555	Goat	1:2000	Thermo Fisher Scientific (Waltham, MA, USA)
β3-tubulin	Rabbit	1:100	Abcam (Cambridge, UK)
S100b	Mouse	1:100	Invitrogen (Carlsbad, CA, USA)

## Data Availability

All data can be found in the author’s official preclinical trial documents.

## Supplementary Materials

The following supporting information can be downloaded at https://www.mdpi.com/article/10.3390/cimb46080507/s1, Figure S1: Creation of a rat limb ischemia model; Figure S2: Laser flowmetry of blood flow intensity in intact and ischemic hind limbs on the 14th day of the experiment; Figure S3: Immunohistochemical visualization of CD31+ cells in the distal part of calf muscle at 28 dpi; Figure S4: Immunohistochemical visualization of CD31+ cells in the distal part of the calf muscle at 42 dpi.
