# Supplementary material for: The Combined Delivery of the Vegf, Ang, and Gdnf Genes Stimulates Angiogenesis and Improves Post-Ischemic Innervation and Regeneration in Skeletal Muscle
Source: Curr Issues Mol Biol. 2024 Aug 5;46(8):8611–26. doi: 10.3390/cimb46080507 (PMC11352388; doi:10.3390/cimb46080507)
Supplement: Supplementary file 1 [file cimb-46-00507-s001.zip › cimb-3088062-supplementary.pdf]

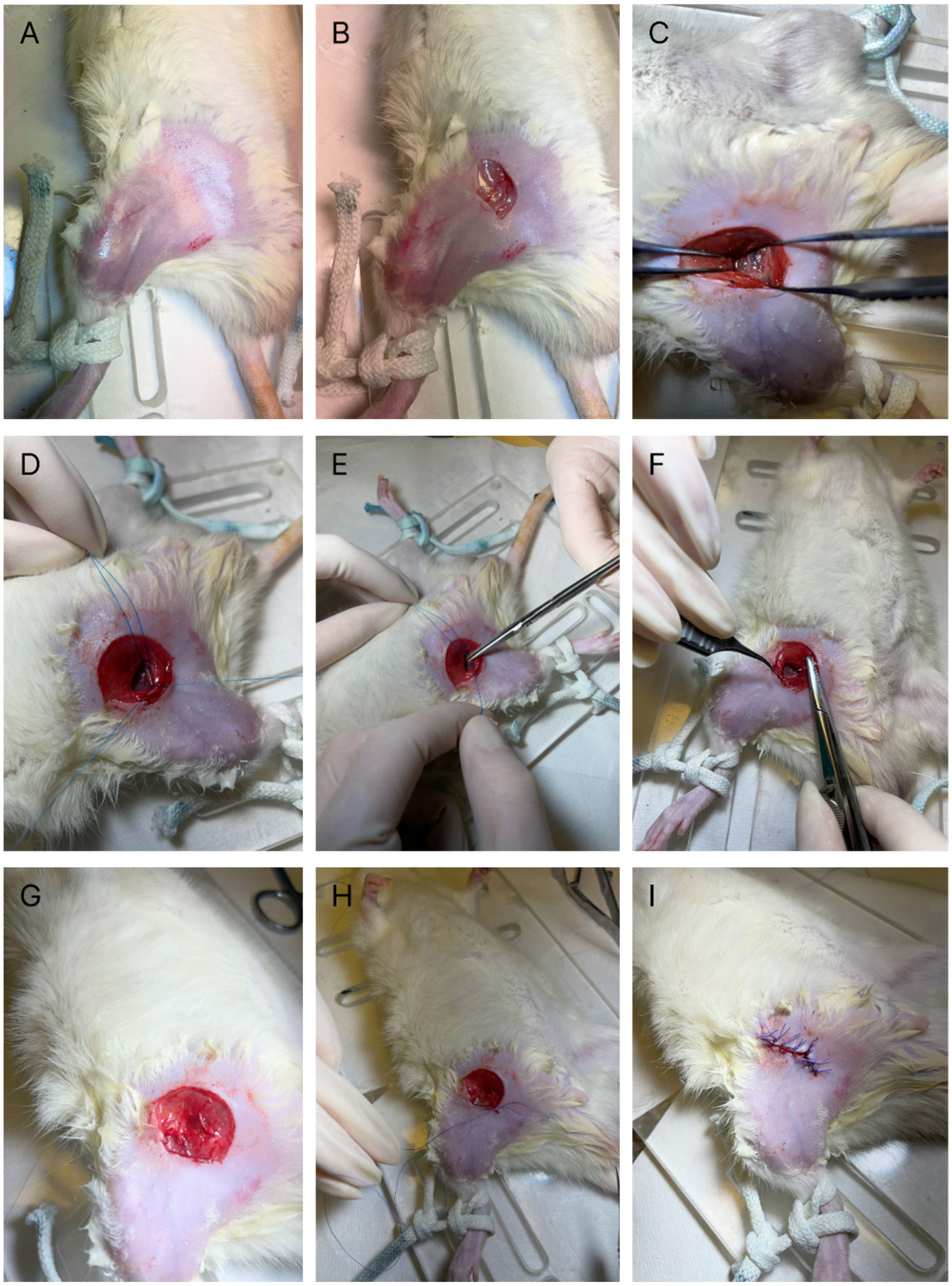

Suppl. Figure S1. Creation of a rat limb ischemia model by ligation and subsequent resection of the femoral artery. A-preparation of the operating field. B-Skin incision is made. C- isolation of the femoral artery. D ligatures are applied proximally and distally to the femoral artery. E section of the artery between the ligatures is dissected. F-layer suturing of the wound, G-fascia sutured, H-overlapping cutaneous sutures. I- the wound is completely sutured.

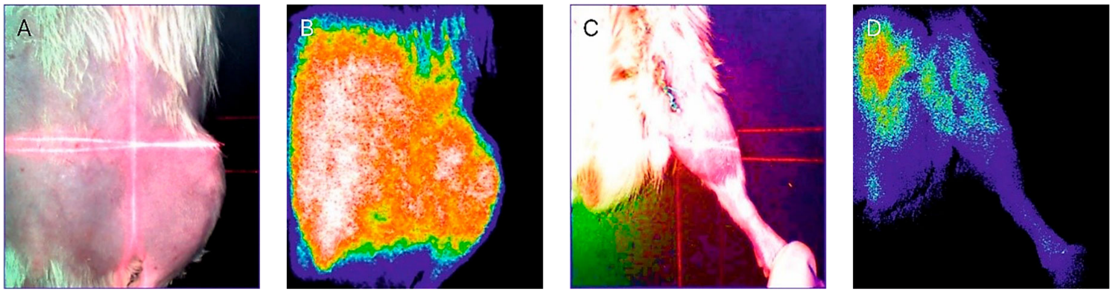

Suppl. Figure S2. Laser flowmetry of blood flow intensity in intact (A, B) and ischemic (C, D) hind limbs on the 14th day of the experiment. A, C - mode of beam focusing on the measurement area, B, D - laser flowmetry mode. The beams correspond to the area of blood flow measurement; Fig. B shows more pronounced blood flow in the limb.

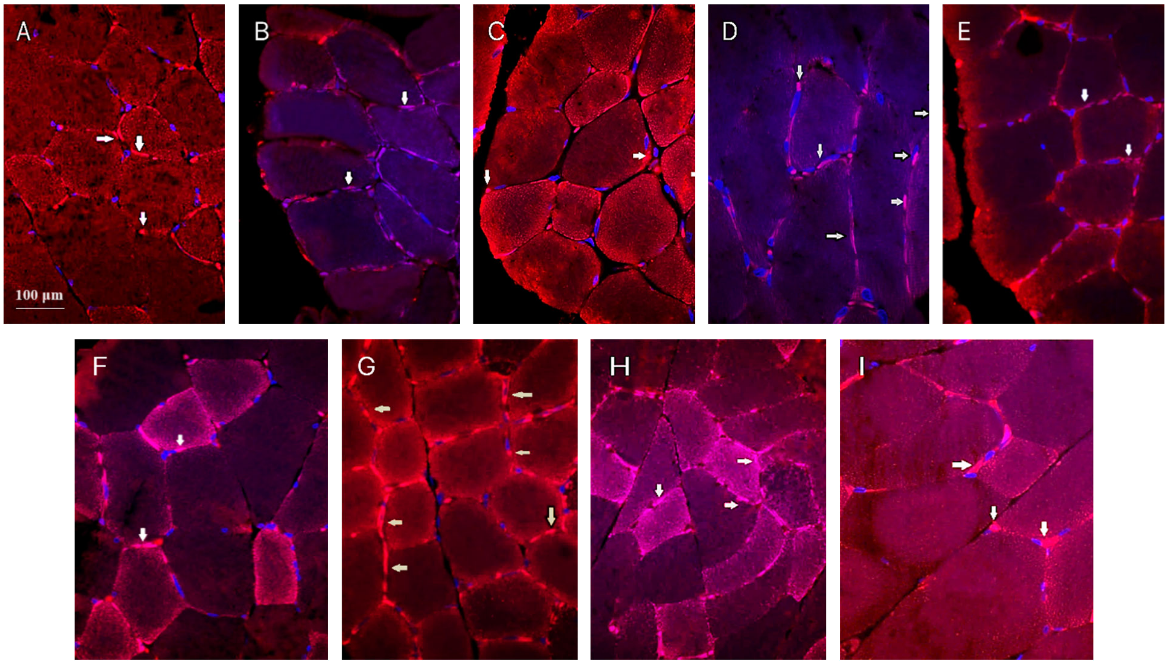

Suppl. Figure S3. Immunohistochemical visualization of CD31+ cells (arrows) in the distal part of calf muscle at 28 dpi in control (A) groups, Ad5-*Vegf* (B), Ad5-*Ang* (C), Ad5-*Vegf*+Ad5-Ang (D), Ad5-*Vegf*+Ad5-Ang+Ad5-Gdnf (E), UCBC Ad5-*Vegf* (F), UCBC Ad5-Ang (G), UCBC Ad5-*Vegf*+*Ad5-Ang*(H), UCBC Ad5-*Vegf*+Ad5-*Ang*+Ad5-Gdnf(I). Confocal microscopy. Nuclei stained with DAPI (blue).

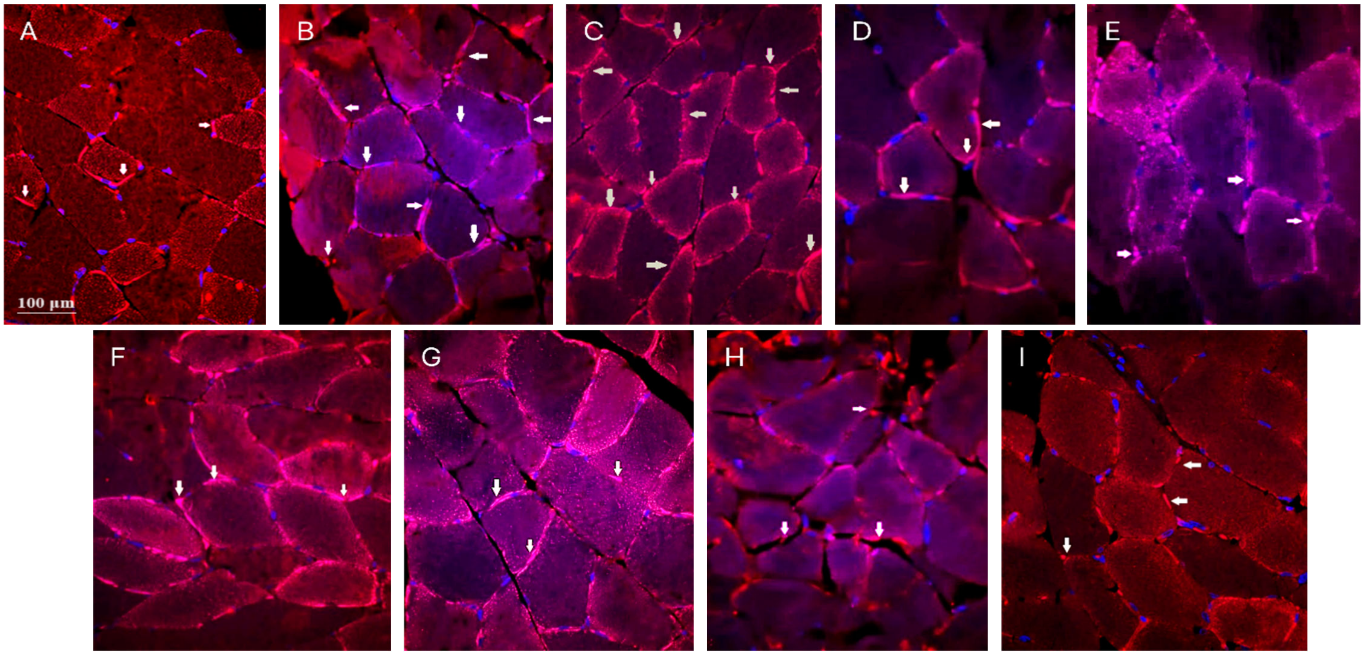

Suppl. Figure S4. Immunohistochemical visualization of CD31+ cells (arrows) in the distal part of the calf muscle at 42 dpi in control (A) groups, Ad5-*Vegf* (B), Ad5-*Ang* (C), Ad5-*Vegf*+Ad5-*Ang* (D), Ad5-*Vegf*+Ad5-*Ang*+Ad5-*Gdnf* (E), UCBC Ad5-*Vegf* (F), UCBC Ad5-*Ang* (G), UCBC Ad5-*Vegf*+Ad5-*Ang*(H), UCBC Ad5-*Vegf*+Ad5-*Ang*+Ad5-Gdnf(I). Confocal microscopy. Nuclei stained with DAPI (blue).
